# Targeting folate receptor alpha for cancer treatment

**DOI:** 10.18632/oncotarget.9651

**Published:** 2016-05-27

**Authors:** Anthony Cheung, Heather J. Bax, Debra H. Josephs, Kristina M. Ilieva, Giulia Pellizzari, James Opzoomer, Jacinta Bloomfield, Matthew Fittall, Anita Grigoriadis, Mariangela Figini, Silvana Canevari, James F. Spicer, Andrew N. Tutt, Sophia N. Karagiannis

**Affiliations:** ^1^ St. John's Institute of Dermatology, Division of Genetics and Molecular Medicine, Faculty of Life Sciences and Medicine, King's College London & NIHR Biomedical Research Centre at Guy's and St. Thomas' Hospitals and King's College London, Guy's Hospital, King's College London, London, United Kingdom; ^2^ Breast Cancer Now Research Unit, Faculty of Life Sciences and Medicine, Guy's Hospital, King's College London, London, United Kingdom; ^3^ Division of Cancer Studies, Faculty of Life Sciences and Medicine, Guy's Hospital, King's College London, London, United Kingdom; ^4^ Unit of Molecular Therapies, Department of Experimental Oncology and Molecular Medicine, Fondazione IRCCS Istituto Nazionale dei Tumori, Milan, Italy

**Keywords:** folate receptor alpha, cancer, biomarker, monoclonal antibodies, immunotherapy

## Abstract

Promising targeted treatments and immunotherapy strategies in oncology and advancements in our understanding of molecular pathways that underpin cancer development have reignited interest in the tumor-associated antigen Folate Receptor alpha (FRα). FRα is a glycosylphosphatidylinositol (GPI)-anchored membrane protein. Its overexpression in tumors such as ovarian, breast and lung cancers, low and restricted distribution in normal tissues, alongside emerging insights into tumor-promoting functions and association of expression with patient prognosis, together render FRα an attractive therapeutic target. In this review, we summarize the role of FRα in cancer development, we consider FRα as a potential diagnostic and prognostic tool, and we discuss different targeted treatment approaches with a specific focus on monoclonal antibodies. Renewed attention to FRα may point to novel individualized treatment approaches to improve the clinical management of patient groups that do not adequately benefit from current conventional therapies.

## INTRODUCTION

Water soluble vitamin B9 can occur as ‘folate’ (enriched in dark leafy vegetables) and as ‘folic acid’ (a synthetic folate compound used as a vitamin supplement). A sufficient intake of folate is needed in rapidly proliferating cells for the one-carbon metabolic reaction and DNA biosynthesis, repair and methylation [1]. Dysregulated folate metabolism has been associated with embryonic developmental disorders, cardiovascular disease and brain defects [2–4]. Folate is transported across the cellular membrane in three ways. The main route of uptake is through the reduced folate carrier (RFC), which is ubiquitously distributed and aids the uptake of dietary folate [5]. The second route is through the proton-coupled folate transporter (PCFT), which utilizes the transmembrane proton gradient to mediate folate transport into the cells [6]. Finally, folate can be transported by folate receptors, of which there are four glycopolypeptide members (FRα, FRβ, FRγ and FRδ), with molecular weights ranging from 38 to 45 kDa [7]. The alpha isoform, Folate Receptor α (FRα), also known as FOLR1 or folate binding protein (FBP), is a glycosylphosphatidylinositol (GPI)-anchored membrane protein with high affinity for binding and coordinating transport of the active form of folate, 5-methyltetrahydrofolate (5-MTF) [8, 9].

FRα has been reported to be overexpressed in solid tumors such as ovarian, lung and breast carcinomas [10–12]. On the other hand, the distribution of FRα in normal human tissues is restricted to low level expression in the apical surfaces of some organs such as the kidney, lung and choroid plexus [9]. Studies have demonstrated that overexpression of FRα may render a growth advantage for cancer cells through mechanisms both relating to, as well as being independent of, folate uptake [13, 14].

Here, we review FRα as a folate carrier and a tumor-associated signalling molecule in relation to its potential as a target, and as a diagnostic and prognostic tool, in oncology. We describe examples of past and present treatment approaches and promising therapeutic avenues, with specific focus on monoclonal antibodies for ovarian, lung and breast cancer, as examples of FRα-positive malignancies.

## FRα-MEDIATED INTERNALIZATION OF FOLATES AND REGULATION OF CANCER SIGNALING

Traditionally FRα is described as a transporter to internalize folate. Folate trafficking *via* FRα is thought to occur by a non-classical lipid raft-mediated endocytosis pathway, namely potocytosis, which does not involve clathrin-coated pits. This pathway is associated with caveolae vesicles [15]. Folate binds specifically to FRα creating a receptor-ligand complex; then through invagination and budding off, intracellular vesicles are formed. Once internalized, the vesicles uncoat and single vesicles join together forming early endosomes, which undergo acidification and subsequent fusion with lysosomes to release folates for the one-carbon metabolic reaction [16, 17].

FRα overexpression in different solid tumors can potentially contribute to cancer development in different ways. A number of studies have suggested parallel roles of FRα in both cell growth regulation and signaling functions. Boshnjaku *et al.* reported that following folate uptake and internalization, FRα can then translocate to the nucleus and act as a transcription factor, binding to cis-regulatory elements. Through this mechanism, FRα may directly regulate the expression of key developmental genes in cancer cells [18]. Ovarian cancer cells transfected with a single-chain intrabody targeting FRα showed reduced cell surface expression and subsequent impaired tumor cell proliferation, reduced colony formation, and dysregulated adhesion; together signs of reversing tumor cell transformed phenotype [19]. Furthermore, folate uptake can promote cancer cell proliferation, migration and loss of adhesion through downregulation of the cell-cell adhesion molecule, E-cadherin, promoting cellular motility and metastasis. In concordance, it was also reported that the absence of E-cadherin expression correlated with decreased patient survival in ovarian carcinomas [20]. FRα knockdown in ovarian carcinoma cell lines could inhibit folate-mediated cell proliferation and suppress an invasive phenotype [14]. FRα has also been demonstrated to inhibit caveolin-1, thereby supporting anchorage-independent growth and proliferation of tumor cells and promoting cancer progression [21, 22].

More recently, FRα has been demonstrated to contribute to cancer malignancy by acting as a signaling molecule. Similarly to other GPI family proteins, FRα is thought to initiate intracellular regulatory signaling networks upon binding with folate. FRα overexpression has been reported to be associated with increased STAT3 signaling [23]. Hansen *et al.* demonstrated that folate binding to FRα could induce STAT3 activation *via* a GP130 co-receptor-mediated JAK-dependent process [24]. Moreover, phosphorylated LYN tyrosine kinase was found in anti-FR mAb precipitates of FRα-expressing tumor cell lysates [25]. These findings suggest the receptor has the potential to form macromolecular complexes in which FRα can trigger intracellular signaling. The basal subtype of breast cancers have been observed to express high levels of LYN, which has been reported to regulate the phosphorylation of a non-receptor tyrosine kinase, PEAK1, to promote ERK and STAT3 activation, as well as to support cellular transition to a mesenchymal phenotype, increasing cell motility and invasion [26]. Furthermore, FRα overexpression is frequently reported to be expressed in metastatic foci and recurrent tumors [27], even in microenvironments with limited folate availability.

**Figure 1:**
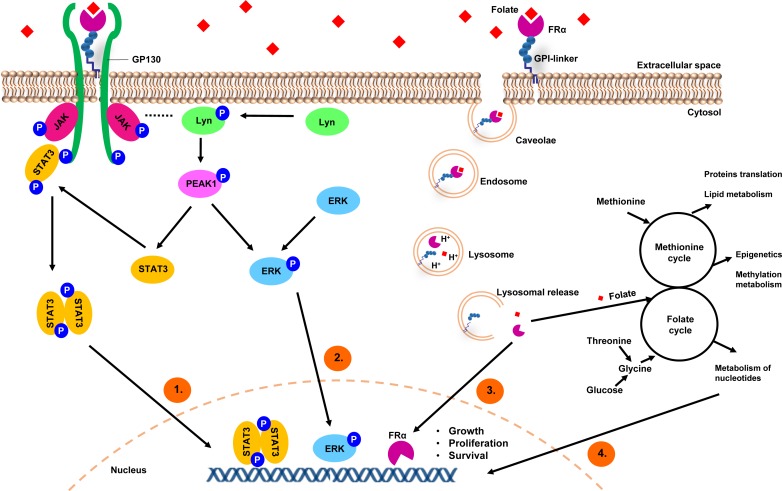
A model depicting FRα-mediated internalization of folates and regulation of cancer signaling 1) Folate binding to FRα could induce STAT3 activation *via* a GP130 co-receptor mediated JAK-dependent process. 2) FRα may form macromolecular complexes with LYN tyrosine kinase, which has been reported to regulate the phosphorylation of PEAK1 to promote ERK and STAT3 activation. 3) GPI-anchored FRα is internalized in caveolae vesicles and forms early endosomes, which undergo acidification and subsequent fusion with lysosomes to release FRα and folate. FRα is then translocated to the nucleus and acts directly as a transcription factor. 4) FRα acts as a folate transporter; a sufficient intake of folate is needed in rapidly proliferating cells for the one-carbon metabolic reaction and DNA biosynthesis, repair and methylation.

Together, these studies strongly suggest that FRα may function not only as a folate transporter, but may also confer signaling and growth advantages on malignant cells (as depicted in Figure 1) [28].

## FRα: A THERAPEUTIC TARGET, DIAGNOSTIC AND/OR PROGNOSTIC TOOL FOR THE MANAGEMENT OF SOLID TUMORS

### FRα expression in non-malignant tissues

FRα expression has been examined by several methods including immunohistochemistry using monoclonal antibodies, folate ligand binding assays, measurement of mRNA levels by qPCR, and by flow cytometry [8, 29–31]. Expression of FRα in normal tissues is restricted to the luminal surface of the kidney, intestine, lung, retina, placenta and choroid plexus [30, 32, 33]. Importantly, in all normal tissues except the kidneys, the receptor is confined to the apical surface of the epithelium that is out of direct contact with folate and any folate receptor-targeting agents in the circulation [34, 35]. The FRα expressed in the kidneys functions as a salvage receptor to retrieve and prevent loss of folate in the urine. However, folate is not retained in the kidneys, and as such no lethal toxicities have been observed in rodent or human subjects treated with FRα-targeting agents [35, 36].

### FRα expression in epithelial tumors

**Figure 2:**
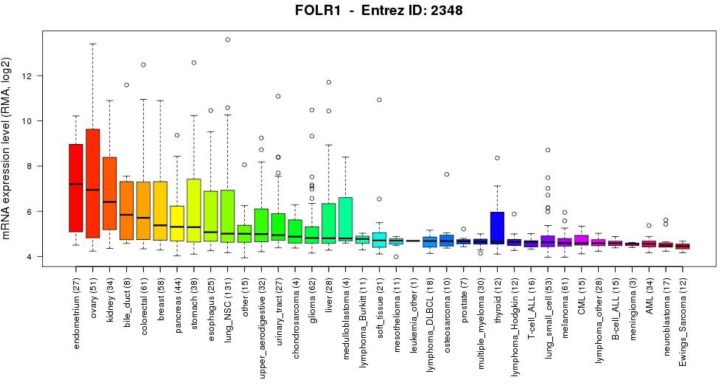
FOLR1 gene expression levels in cancer models A summary of gene expression data (generated from the Cancer Cell Line Encyclopaedia) showing the levels of mRNA expression for the FOLR1 gene on a logarithmic scale in various cancer cell types. (parentheses show number of cell lines per tumor type)

Cancers found to overexpress FRα are often of epithelial origin, including cancers of the ovary, breast, pleura, lung, cervix, endometrium, kidney, bladder and brain [29, 37]. FOLR1 mRNA expression levels for various types of cancer have been studied (expression levels in cell line panels summarized in Figure 2) [38].

#### Ovarian cancer

In ovarian cancer, FRα is overexpressed in 80% of epithelial ovarian cancers (EOCs) and expression has been shown to significantly correlate with histological grade and stage [10, 14]. The expression of FRα is considered a marker of tumor aggressiveness, and although there is conflicting data when all ovarian carcinoma histotypes are considered, elevated FRα expression is associated with lower disease-free interval (DFI) and poor overall survival (OS) in patients with disease of serous origin [27, 39].

FRα is also thought to facilitate resistance to chemotherapy in ovarian carcinoma patients, with higher tissue FRα expression associated with lower response rate to chemotherapeutic agents [28, 39, 40]. Nevertheless, a number of studies have shown that FRα tumor surface expression does not differ between paired samples before and after chemotherapy. This suggests that chemotherapeutic agents do not affect antigen expression and FRα-positivity can be used to detect recurrent disease [41, 42] and strengthen the rationale for the potential utility of FRα-targeting therapeutics for both newly diagnosed and recurrent disease.

#### Lung cancer

Non-small cell lung cancer (NSCLC) accounts for 80% of lung cancers. Despite advances in surgical techniques, radiotherapeutic approaches and chemotherapy regimens, and the development of novel molecular targeted agents, the prognosis is poor with a 5-year all-stage survival rate of 21% [11]. Both common subtypes of NSCLC, adenocarcinoma and squamous cell carcinomas (SCCs), to a lesser degree, have been reported to express high levels of FRα [43–46]. The level of FRα expression by tumor cells was shown to be associated with improved OS in patients with resected adenocarcinomas [46]. Similarly, in another study, higher FRα expression levels were observed in well-differentiated, earlier stage lung tumors compared to more poorly-differentiated tumors from advanced stage adenocarcinoma patients, and higher FOLR1 gene expression levels correlated with significantly higher 3-year disease free survival (DFS) and OS rates in this cohort as well [44]. Most recently, the validity of FRα-targeting therapies, has been strengthened by observed concordance of high FRα levels between biopsies, primary tumors and metastases in these patients [11].

Overexpression of FRα has also been detected in up to 72% of patients with another uncommon and aggressive form of thoracic cancer, pleural mesothelioma [47, 48]. However, more studies are required to evaluate the diagnostic and prognostic significance of tumor FRα expression in mesothelioma.

#### Breast cancer

A number of studies have reported overexpression of FRα in breast cancers [12, 49]. Most recently, high FRα tissue expression was observed in 74% of estrogen receptor (ER)/progesterone receptor (PR)-negative breast cancers [13, 50–52]. Steroid hormones mediate physiologically normal FRα regulation; in particular, oestrogen has been found to downregulate the expression of FRα. This has been demonstrated in studies that report a negative correlation between tumors that express ER and those which express FRα [13, 52]. Supporting this notion, drugs such as tamoxifen, which bind and inhibit ER function, can also cause a rise in FRα expression [53]. Triple negative breast cancer (TNBC), which is characterized by the lack of ER, PR and human epidermal growth factor receptor 2 (HER2), represent 10-15% of all breast carcinomas. Despite representing a minority of breast cancer cases, this particular subtype accounts for a disproportionate number of cases of metastatic disease and deaths, due to its more aggressive natural history and lack of available targeted therapies [54, 55]. High FRα tissue expression was observed in 80% of TNBCs, making it an attractive therapeutic target in this breast cancer type [51].

FRα expression by breast carcinomas, including TNBCs, is significantly associated with high histologic grade and advanced stage, and high proliferative activity as determined by expression of Ki-67. Overexpression of FRα was also significantly associated with poorer disease-free survival [49, 52]. In addition, correlation of high FRα expression between primary tumors and local and distant metastases has been observed, suggesting that most metastatic disease could potentially be treated with anti-FRα therapies when the primary tumor shows overexpression.

Overall, these studies, across a number of cancer cell types, suggest that targeting this tumor-associated antigen may offer clinical benefit to patients with unmet need for novel therapeutic options. The low and restricted distribution of FRα in normal tissues, alongside the presence of other folate transporters, which can process folates, may suggest that targeting FRα would not restrict all folate uptake, and may not affect normal cell survival or proliferation nor impair normal tissue homeostasis. Overexpression of FRα in the blood-accessible basal and lateral membranes of epithelial carcinomas, and suggested contributions to tumor cell growth and proliferation, together point to targeting FRα as an attractive and potentially non-toxic treatment approach.

### Soluble FRα

Membrane-associated FRα can also be released by proteolytic cleavage with membrane-associated protease and GPI-specific phospholipases [9, 56]. FRα is detected as a soluble form in the sera of some cancer patients by a number of techniques, including microfiltration, immunoblotting, electrochemiluminescence, and ELISA [23, 57–59]. Soluble FRα (sFRα) is reported to be low or undetected in normal human sera [23, 58]. A number of studies of the serum from patients with both serous and non-serous ovarian carcinomas have demonstrated elevated levels of sFRα, compared to healthy individuals. Importantly for the potential use of sFRα as a diagnostic biomarker, elevated levels of sFRα were observed even in early stage I/II disease [23, 59]. Furthermore, levels of sFRα have been reported to correlate with FIGO stage, histological types and tumor grade [57, 59]. Most recently, in a cohort of 221 patients, the prognostic significance of sFRα was supported by findings that patients with lower sFRα levels had significantly longer progression-free survival (PFS) than those with high serum levels. A trend in increasing sFRα and tumor expression levels measured by immunohistochemistry was also reported, suggesting that sFRα may offer a minimally-invasive alternative to predict local tumor FRα positivity and may present a surrogate marker for FRα tumor expression [57].

Studies are still needed to examine sFRα levels in the serum of patients with breast and lung cancers in order to evaluate its potential as a diagnostic and prognostic biomarker across cancer types and in those individuals where other clinically-used serum biomarkers are absent or unhelpful in monitoring or predicting disease progression. In addition, studies are required to evaluate the potential inhibitory impact of high sFRα levels on the efficacy of FRα-targeting therapies.

## CLINICAL APPLICATION OF FRα-BASED AGENTS

**Figure 3:**
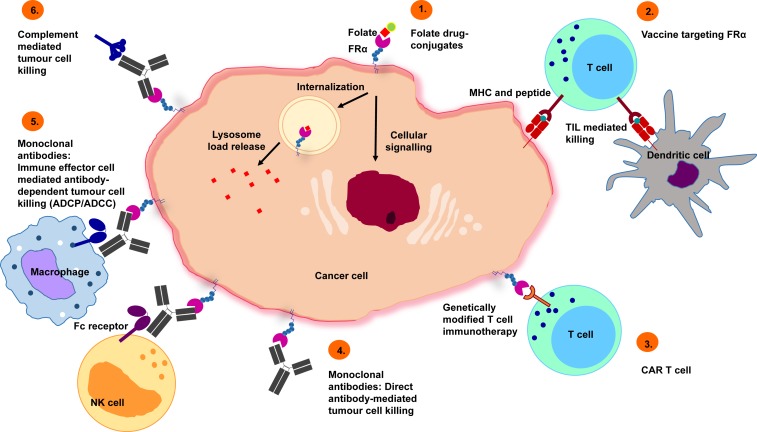
Potential treatment approaches targeting FRα 1) Folate drug-conjugates: chemotherapeutic agents, such as vintafolide, have been conjugated to folate for FRα targeting. 2) Vaccines targeting FRα: autologous dendritic cells engineered with FRα mRNA commence an anti-FRα immune response, mediated by T-cells. 3) Chimeric antigen receptor (CAR) T cells: CAR T cells recognizing FRα trigger tumor cell killing. 4) Monoclonal antibodies (direct effects): Specific recognition of FRα lead to inhibition of downstream signaling events that cause tumor cell death. 5) Monoclonal antibodies (immune effector cell engagement): antibodies link FRα-expressing tumor cells with immune effector cells that bear Fc receptors, potentiate effector cell activation and target-neutralizing functions by engendering antibody-dependent effector cell-mediated cytotoxicity (ADCC), phagocytosis (ADCP) or 6) complement-dependent cytotoxicity (CDC) activation.

A range of FRα-targeting approaches, including folic acid derivatives, folate drug-conjugates and small molecules, vaccines, T-cell therapies and monoclonal antibodies, have been developed for clinical application for both imaging and therapeutic purposes (summarized in Figure 3).

### FRα-targeted imaging

Although there is evidence to suggest that FRα-positive tumors can be detected by radiolabelled antibodies [60, 61], the use of antibodies as diagnostic or prognostic tools may be limited. Poor contrasts between tumor and non-target tissue are often achieved following prolonged circulation times and slow uptake by tumors [62].

Imaging of radiolabelled derivatives of folic acid may offer greater tissue selectivity. Folic acid derivatives conjugated to a variety of metal chelates have allowed non-invasive detection of FRα-positive tumors in both animal models and patients [62]. Of these probes, the most extensively studied is ^111^In-DPTA-folate, which demonstrated good on-target to off-tumor contrast in mouse models, leading to a phase I/II trial. Patients with newly diagnosed or suspected recurrent ovarian cancer were imaged by single-proton emission computed tomography (SPECT) using this agent. Newly diagnosed malignancies were detected at a sensitivity of 100% (*n* = 7), although detection of recurrent disease was more problematic. Overall, this agent appeared to be safe, and possibly an effective diagnostic tool, although this evaluation was limited by the small sample size of 33 patients [63]. Another folic acid peptide derivative (EC20) conjugated with ^99m^Technetium (^99m^Tc-EC20), which is less costly and has a shorter half-life than ^111^In-DPTA-folate, has also demonstrated promising qualities in preclinical models of ovarian cancer [64]. Furthermore, this agent has demonstrated superior FRα affinity and clearance from the blood and kidneys, and has been used to image > 200 patients to date, without any imaging-related adverse events reported [36].

By contrast, using macromolecular dendrimer polychelates conjugated to folic acid in magnetic resonance imaging (MRI) showed improved tumor to non-target contrast in mouse models of ovarian cancer, with specific accumulation in the tumor. This is thought to be due to a reduction in microcapillary permeation by the large probe molecule, and thus a greater relative accumulation in FRα-positive tumors [65]. A more recent study demonstrated the clinical promise of FRα-specific ultrasmall superparamagnetic iron oxides in MRI of a rat model of breast cancer. This agent was specifically retained in FRα-positive breast tumors and may also be useful as a non-invasive tool to diagnose and discriminate FRα-positivity [66].

Agents comprising folic acid conjugated to fluorescent dyes have been evaluated. These agents provide good contrast between malignant and healthy tissues in animal models. However, due to the optical qualities of the dyes, surgical opening of the cancer site was required to obtain images. In future it may be possible to use such agents in cutaneous or subcutaneous cancers, or to guide endoscopic or open surgery for cancer [36].

Overall, these imaging agents may allow clinical detection of FRα-positive tumors, which may aid diagnosis and disease surveillance. However, improvements in sensitivity and future studies to evaluate prognostic utility are needed to validate this approach as a clinical tool.

### Etarfolatide

Etarfolatide is a ^99m^Tc-based imaging agent, also known as FolateScan, which has been the subject of many studies in patients with ovarian, kidney, lung cancer and other refractory solid tumors (NCT01686256, NCT01684098) [67]. The purpose of etarfolatide is use as a companion agent with vintafolide, to enable pre-selection of patients with tumors expressing FRα. The administration of folic acid prior to etarfolatide infusion was shown to improve SPECT images, and a phase I clinical study was performed to investigate the safety and pharmacokinetics of etarfolatide up to 7 days following folic acid injection [68]. Etarfolatide has since been used to evaluate the tumor FRα expression of individuals in a number of trials (NCT00511485, NCT01577654 and NCT01999738).

### Folate drug-conjugates

The first folate-conjugated cytotoxic agent to be evaluated in tumor therapy was a maytansinoid conjugate [69]. Since then, a series of chemotherapy agents has been conjugated to folate for FRα targeting, with varying degrees of success. Key clinical trials are summarized in Table 1 [70].

**Table 1:** Key clinical trials of FRα-drug conjugate therapeutics

Drug Name	Alternative Name(s)	Tumor Type	Trial Design	Efficacy Outcome	Safety Outcome	Imaging Outcome	Reference/Trial Number
Vintafolide	EC145 Folate-conjugated DAVLBH	Platinum-resistant ovarian cancer	Randomized phase II trial of Vintafolide + PLD *vs.* PLD alone in platinum-resistant ovarian cancer (PRECEDENT)	Vintafolide demonstrated significantly improved clinical activity compared with PLD alone. Median PFS was 5.0 and 2.7 months for vintafolide + PLD and PLD-alone arms, respectively (*P* = 0 .031). Greatest benefit observed in patients with 100% FR+ lesions, with median PFS 5.5 *vs.* 1.5 months for PLD alone (P = 0.013)	Vintafolide + PLD was well tolerated. Frequency of leukopenia, neutropenia, abdominal pain, and peripheral sensory neuropathy was higher in vintafolide + PLD *vs*. PLD arm	N/A	[77] NCT00722592
		FR+ platinum-resistant ovarian cancer	Phase III study of vintafolide + PLD *vs*. PLD alone in patients with FR+ platinum-resistant ovarian cancer (PROCEED).	At a prespecified interim data analysis, cessation of the study was recommended because vintafolide + PLD *vs*. PLD alone did not meet the prespecified criteria for PFS	No safety concerns were detailed	N/A	[28] NCT01170650
		FR+ NSCLC	Randomized, open-label, phase II trial of vintafolide as second-line treatment *vs*. vintafolide + docetaxel *vs*. docetaxel alone in patients with FR+ NSCLC (TARGET)	Preliminary data for vintafolide + docetaxel showed improvement across all efficacy endpoints *vs*. docetaxel alone. The best improvement was observed in the predefined adenocarcinoma patient subgroup	The safety profile was manageable and consistent with the AEs observed with both therapies	N/A	[75, 76] NCT01577654
EC0225	Folate–desacetylvinblastine-hydrazide folate–mitomycin C	Solid tumors	Phase I study of EC0225 in patients with solid tumors (refractory or metastatic)	Disease stabilization (≥ 4 months) was observed in 26/63 patients	EC0225 was well -tolerated at doses ≤2.3 mg/m^2^. Most frequent AEs included anemia, constipation, leukopenia, fatigue. G3 hypotension and G4 neutropenia occurred in 1 patient each at the highest dose tested (2.88mg/m^2^) defining the MTD	N/A	[79 NCT00441870
Epofolate	BMS-753493 Folate-conjugated epothilone A	Solid tumors	Phase I study of epothilone folate (BMS-753493) in patients with advanced solid tumors	Best overall response was SD in 19% patients. Median duration of response was 85 days. No correlation between FR status and response. Interim analysis indicated that the benefit risk profile of BMS-753493 was not favorable and did not support further investigation of this agent	The MTD was reached. DLTs included ALT and AST elevations, diarrhoea, nausea, fatigue, oesophagitis and mucosal inflammation	N/A	[80, 81] NCT00546247
EC0489	Folate–desacetylvinblastine hydrazide with modified linker	Solid tumors	Phase I study of EC0489 in patients with refractory or metastatic tumors	Not reported	Patients treated at 2.5mg/m^2^ experienced toxicities characteristic of vinca alkaloids (e.g., mild neuropathy), but not significant constipation nor gastrointestinal-related toxicity	N/A	[82] NCT00852189
EC1456	Folate–tubulysin	TNBC, advanced NSCLC and ovarian cancer	Phase I dose-escalation study of EC1456 (Part A) including a study of efficacy in patients with TNBC, advanced NSCLC and ovarian cancer treated at the MTD (Part B)	Data awaited	Data awaited	N/A	NCT01999738

AE, adverse event; ALT, alanine aminotransferase; AST, aspartate aminotransferase; DLT, dose-limiting toxicity; G, CTCAE grade; MTD, maximum tolerated dose; NSCLC, non-small cell lung cancer; PFS, progression-free survival; PLD, pegylated liposomal doxorubicin; SD, stable disease; TNBC, triple negative breast cancer. Clinical trial number (NCT) available from: http://clinicaltrials.gov.

### Vintafolide and other folate conjugates

Vintafolide, or EC145, is a folate conjugate of desacetylvin-blastinemonohydrazide (DAVLBH), a derivative of the microtubule destabilizing agent vinblastine [71]. Following receptor-mediated endocytosis, the self-immolative disulphide-linker system enables the release of DAVLBH into the tumor cell endosome, leading to inhibition of cell division and induction of cell death [72, 73]. The safety of vintafolide was first assessed in a phase I clinical study in various solid tumors [74], while multiple phase II and III clinical trials have been conducted in lung and ovarian cancer (NCT00507741, NCT01577654, NCT00722592, NCT00511485 and NCT01170650) [69].

One clinical study, EC-FV-03 (NCT00511485), consisted of a single-arm phase II trial that assessed vintafolide as a single agent in patients with lung adenocarcinoma, who had previously been treated with at least two chemotherapy regimens. Analysis of survival outcomes demonstrated superior median PFS in patients with high FRα tumor expression compared with those with low expression. The median OS also showed a trend towards improvement [75], although the non-randomized study design precluded drawing any firm efficacy conclusion given the likely prognostic value of FR-alpha expression. The randomized, open-label, phase II TARGET, study (NCT01577654), has also recruited patients with FRα-positive non-small cell lung cancer. Preliminary results for vintafolide combined with docetaxel suggest improvement across all efficacy endpoints over docetaxel as single agent in the control arm [76].

Among the multiple clinical studies of vintafolide conducted on ovarian cancer patients, of particular interest is the open-label phase II PRECEDENT study (NCT00722592) [77]. In this trial, patients who had received more than two previous chemotherapeutics were randomized to receive vintafolide and PEGylated liposomal doxorubicin (PLD) or PLD alone until progression or death. Median PFS, compared to PLD treatment alone, was most superior in individuals with higher FRα expression. This was the first combination therapy to suggest a significant prolongation of PFS over standard therapy in platinum-resistant ovarian cancer patients [28]. The fact that FRα-negative patients (based on etarfolatide imaging) did not benefit from vintafolide and PLD combination therapy, whereas patients with highly FRα-positive tumors did, supported the strategy of using an imaging agent to identify the FRα-positive patient population for FRα-targeted therapy. However, the subsequent phase III PROCEED trial, designed to further evaluate the clinical efficacy of vintafolide combined with PLD (NCT01170650) was discontinued at an interim analysis because the experimental arm did not meet the pre-specified primary outcome for PFS improvement required [78].

Following vintafolide, several additional folate-conjugated agents have been studied. EC0225, a folate conjugated to both a vinca alkaloid and mitomycin, was evaluated in a phase I trial which determined the maximum tolerated dose (MTD) (NCT00441870) [79]. Results are awaited from a phase I/II trial (NCT00546247) of BMS-753493 (Epofolate), a folate conjugate of epothilone A, which is a microtubule stabilizing agent [80, 81]. EC0489, an analogue of vintafolide, has been shown to have reduced hepatic clearance [82] and its MTD was evaluated in a phase I trial of metastatic tumors (NCT00852189). EC1456, a folic acid-tubulysin small-molecule drug conjugate, is currently undergoing phase I trial in patients with advanced NSCLC, ovarian cancer and TNBC (NCT01999738). IMGN853, an anti-FRα mAb conjugated with the maytansinoid, DM4, is now being evaluated in a number of phase I clinical trials to evaluate its safety and pharmacokinetics/pharmacodynamics alone (NCT01609556), and in combination with chemotherapy and bevacizumab (NCT02606305), in patients with EOC and other FRα-positive tumors. Furthermore, a phase II trial, to compare the efficacy of IMGN853 to standard chemotherapies, is recruiting patients with FRα-positive tumors (NCT02631876). Folate-conjugated carboplatin was not considered a promising therapy due to neutralization by folate receptor-mediated endocytosis [83], but several other microtubule poisons have shown moderate promise in *in vitro* and *in vivo* models [84, 85].

Unlike anti-FRα antibodies, folate conjugates target both FRα and the functional form of FRβ. This could potentially enable the targeting of tumors that are low in FRα but high in FRβ, including tumors that are infiltrated by large numbers of tumor-associated macrophages (TAMs), known to express FRβ. Targeting pro-tumoral TAMs with FRβ-specific agents is a widening avenue of research discussed in detail in other publications [86–89].

The bi-specificity of folate drug-conjugates might reduce the selectivity of these drugs for tumor cells, but may potentially expand the range of targets to include elements of the tumor microenvironment. A potential disadvantage of folate-conjugated drug delivery is that binding is competitively-inhibited by excess serum free folate. A further potential issue is the exposure of FRα expressed in the apical membrane of the proximal renal tubules following filtration of low molecular weight (LMW) folate conjugates [90]. While this has not adversely affected clinical translation of folate conjugates, it may affect the clinical application of LMW folate conjugate-based radiopharmaceuticals for targeted radiotherapy.

### Anti-FRα small molecule drugs

Conventional anti-folate drugs, such as pemetrexed and methotrexate, are often carried by the high capacity RFC, which is ubiquitously expressed on normal and tumor cells, leading to non-specific activity and associated reductions in patient tolerability. Therefore, anti-folate thymidylate synthase (TS) and glycinamide ribonucleotide formyl transferase (GARFTase) inhibitors, with negligible affinity for RFC and high-affinity for FRα, have been developed. These anti-FRα small molecules are thought to be more tumor-targeted, due to the restricted expression of FRα on normal tissue on apical membranes away from the bloodstream [91, 92]. Examples of anti-FRα TS inhibitors include CB300638 and ONX-0801. Pre-clinical studies of these agents have demonstrated anti-tumor efficacy in FRα-expressing tumors, and reduced transportation through RFC, which is expected to confer reduced patient toxicity [91, 93, 94]. A limitation for anti-FRα small molecules may be the level of FRα expression by target tumor cells, which needs to be sufficient to transport adequate amounts of the drug into the cell in order to inhibit TS and cause cell death. Much of the pre-clinical studies have used cell lines and therefore future work will be required to determine whether the expression levels of FRα are sufficient for functional effects against patient tumors [95]. A phase I trial of ONX-0801 is now recruiting patients with solid tumors, expected to have high FRα expression, such as EOC patients, in order to evaluate the safety and efficacy of two dosing schedules (NCT02360345).

### Vaccines targeting FRα

Increased immunity to FRα has been reported in patients with FRα-expressing ovarian cancer in comparison to healthy controls, suggesting that this may be a target for cell-based and peptide immunotherapies [23]. Key clinical trials are summarized in Table 2 [70].

A case report of a single patient with ovarian carcinoma, vaccinated with autologous dendritic cells that were engineered with mRNA-encoded FRα, demonstrated a vaccine-induced T cell reactivity, resulting in more than 50% of tumor regression, as well as a reduction of tumor markers levels, suggesting this strategy may hold promise as a cancer treatment [96]. A FRα-targeted hapten immunotherapeutic regimen known as ‘Folate Immune’, which combines folate-targeted vaccine EC90, together with an adjuvant, GPI-0100, and a folate-hapten conjugate EC17, was designed to convert poorly immunogenic tumors to highly immunogenic tumors. Patients first underwent vaccination with EC90 and GPI-0100 adjuvant to stimulate production of specific antibodies, and were then treated with EC17, which is thought to bridge antibodies with FRα-expressing tumor cells and trigger antibody-dependent cellular cytotoxicity and/or phagocytosis (ADCC/ADCP). A phase I study confirmed that this regimen was well tolerated in 33 patients, with mild to moderate injection site reactions being the most common adverse effects reported [97]. A phase II trial was then initiated in 2012to evaluate this therapeutic strategy in patients with metastatic renal cell carcinoma, but has since been terminated due to low patient accrual (NCT00485563).

**Table 2:** Clinical trials of FRα-targeting vaccines and CAR T cells

Drug Name	Alternative Name(s)	Tumor Type	Trial Design	Efficacy Outcome	Safety Outcome	Imaging Outcome	Reference/Trial Number
**Vaccines**							
Folate Immune	EC90/GPI-0100/EC17	Renal cell carcinoma	Phase I study of Folate Immune (EC90 vaccine administered with GPI-0100 adjuvant followed by EC17) in patients with renal cell carcinoma	1/28 (4%) patients had a PR, 15/28 (54%) had SD	Two DLTs seen (G4 anaphylaxis and G3 pancreatitis). Mild - mod injection site reactions were most common AE during vaccination phase. Transient hypersensitivity reactions were most common during treatment phase	N/A	[97] NCT00329368
Folate Immune	EC90/GPI-0100/EC17	Renal cell carcinoma	Phase I study of Folate Immune (EC90 vaccine administered with GPI-0100 adjuvant followed by EC17) in patients with progressive metastatic renal cell carcinoma	Trial terminated due to poor patient accrual		N/A	NCT00485563
Autologous dendritic cells engineered with FRα mRNA	/	FRα+ serous papillary ovarian carcinoma	Single patient report of a patient with serous papillary ovarian carcinoma at second relapse (platinum-resistant) who received a vaccination regimen with autologous dendritic cells engineered with mRNA encoded FRα	CT before treatment and 3 months after the last vaccination (13 months total) demonstrated a PR. CA-125 greatly reduced 4 weeks after the first vaccination and were still at baseline at 11 months after completion of vaccination	Not reported	N/A	[96]
CAR T Cells							
CAR-T cells specific to FRα	/	Ovarian cancer	Phase I study of adoptive transfer of FRα redirected autologous T cells, either with high-dose IL-2 (cohort 1), or followed by immunization with allogeneic peripheral blood mononuclear cells (cohort 2), for recurrent ovarian cancer	No reduction in tumor burden was seen in any patient	5/8 (63%) patients in cohort 1 experienced a G3 – 4 AE	Lack of specific localization of T cells to tumor was observed by tracking ^111^In-labeled adoptively transferred T cells	[133]

AE, adverse event; DLT, dose-limiting toxicity; G, CTCAE grade; PR, partial response; SD, stable disease. Clinical trial number (NCT) available from: http://clinicaltrials.gov.

### Oncolytic virus therapy

Virotherapy is a treatment approach using wild type or genetically engineered viruses [98]. An attenuated measles virus has been evaluated pre-clinically as FRα targeting treatment for ovarian cancer. The anti-tumor activity of oncolytic measles virus highly specific for FRα (MV-αFR) was tested in a xenograft model of ovarian cancer. Treatment of human ovarian cancer-bearing mice with MV-αFR resulted in significant inhibition of tumor growth compared to controls and 50% of mice showed complete regression of tumors. These findings suggested the merit of clinically testing the oncolytic virotherapy approach in FRα-expressing tumors [99].

### Monoclonal antibodies

Monoclonal antibodies (mAbs) can target tumor-associated antigens, such as FRα, on the surface of tumor cells. They can mediate specific anti-tumor activity either by blocking cell signaling or by eliciting immune-mediated cell killing by engaging effector cells or complement. Examples of clinical trials of monoclonal antibodies directed against FRα are summarized in Table 3 [70].

**Table 3:** Clinical trials of FRα-targeting monoclonal antibodies

Drug Name	Alternative Name(s)	Tumor Type	Trial Design	Efficacy Outcome	Safety Outcome	Imaging Outcome	Reference/Trial Number
Farletuzumab	MORab003	Platinum-resistant EOC	Phase I dose escalation study of weekly farletuzumab	Following 1 treatment cycle (4 weeks): No objective responses. SD in 36% patients and CA-125 reduction in 16%	No DLTs. MTD not reached	Radiolabeled tracer studies conducted in 3 patients showed significant tumor uptake	[105] NCT00428766
		Platinum-sensitive EOC	Phase II study of farletuzumab as single agent or in combination with a platinum and taxane in platinum-sensitive, recurrent epithelial ovarian, fallopian tube or primary peritoneal cancer	Farletuzumab alone: 0% normalised CA125, 30% SD as best response, 70% PD. Farletuzumab + chemotherapy: 80.9% normalised CA125. ORR 75%. In 21%, the second progression-free interval was longer than the first	Farletuzumab well-tolerated as single agent, without additive toxicity when administered with chemotherapy. SAEs in 37% patients – 9% considered related to farletuzumab	N/A	[107] NCT00318370
		Platinum-sensitive EOC	Phase Ib safety study of farletuzumab, carboplatin and PLD in patients with platinum-sensitive EOC at first or second relapse	CR in one patient (7%), PRs in 10 patients (67%), SD in 4 patients (27%).	Combination well-tolerated - no farletuzumab-related G3-4 adverse events.	N/A	[106] NCT01004380
		Platinum-sensitive EOC	Phase III double-blind placebo-controlled study of weekly farletuzumab + carboplatin/taxane in platinum-sensitive ovarian cancer at first relapse.	Median PFS 9.0 (placebo), 9.5 (farletuzumab 1.25 mg/kg), and 9.7 (farletuzumab 2.5 mg/kg) months with no statistically significant difference between arms	Most common AEs across arms were those known to be associated with chemotherapy.	N/A	[28] NCT00849667
		Platinum-sensitive recurrent EOC with a low CA125	Phase III global multicenter double-blind randomized placebo-controlled trial of farletuzumab + platinum and a taxane or PLD in patients with first relapse of platinum-sensitive ovarian cancer and a low CA125	Data awaited	Data awaited	N/A	NCT02289950
		Platinum-resistant EOC	Phase III double-blind placebo-controlled study of weekly farletuzumab + paclitaxel in platinum-resistant ovarian cancer at relapse	Predefined criteria for trial continuation were not met.	Trial terminated	N/A	NCT00738699
		FRα+ TNBC with low levels of CA125	Phase II trial of farletuzumab in FRα+ TNBC with low levels of CA125	Data awaited	Data awaited	N/A	[78]
		NSCLC (adenoCa)	Phase II randomized, placebo-controlled, multicenter study of a platinum containing doublet +/− farletuzumab in stage IV adenocarcinoma of the lung	Data awaited	Data awaited	N/A	NCT01218516
MOv18 IgG	/	Ovarian carcinoma	Phase I single infusion dose-escalating study of cMOv18 IgG in patients with primary, residual or recurrent ovarian cancer	Efficacy outcomes not reported	No DLT observed. At doses of ^3^50mg all patients experienced G2 side effects, including fever, headache, and nausea/vomiting	N/A	[112]
^131^I-cMOv18 IgG1	/	Ovarian carcinoma	Kinetics and tissue distribution of ^131^I-cMOv18 IgG1 in patients with ovarian carcinoma	Not reported	Not reported	Good localisation in ovarian carcinoma tissue	[33]
		Ovarian carcinoma	Phase I study of i.p. radioimmunotherapy with ^131^I-mMOv18 IgG1 in patients with ovarian cancer with minimal residual disease	5/16 patients had a CR, 6/16 patients had SD and 5/16 patients had PD	Minimal toxicities observed. One patient mild and transient bone marrow suppression	N/A	[111]
		Ovarian carcinoma	Phase I study of i.p. and i.v. radioimmunotherapy with ^131^I-cMOv18 IgG1 in patients with suspected ovarian cancer scheduled to undergo exploratory laparotomy	Efficacy outcomes not reported	No normal organ toxicity	Tumor uptake in ovarian cancer tissue 3.4% - 12.3% ID/kg for i.p. and 3.6% - 5.4% ID/kg for i.v. administration	[113]
^125^I-, ^123^I-, ^131^I-, cMOv18 IgG1	/	Ovarian carcinoma	Study of simultaneous i.v. and i.p injection of radiolabeled c-MOv18 (using different radionuclides) in patients with ovarian cancer to determine the optimum way to deliver radiolabeled cMOv18	Not reported	No AEs reported	No significant differences found in tumor:normal tissue and tumor:blood ratios for both i.v. and i.p routes. Tumor uptake varied between patients and within same patient	[61]
MOv18 IgE	/	FRα+ advanced tumors	Phase I: First in human study of MOv18 IgE in patients with FRα+ advanced cancer	Data awaited	Data awaited	N/A	NCT02546921

AE, adverse event; cMOv18, chimeric MOv18; CR, complete response; DLT, dose-limiting toxicity; EOC, epithelial ovarian cancer; G, CTCAE grade; *i.p*., intraperitoneal; *i.v.*, intravenous; MTD, maximum tolerated dose; NSCLC, non-small cell lung cancer; ORR, overall response rate; PD, progressive disease; PFS, progression-free survival; PLD, pegylated liposomal doxorubicin; PR, partial response; SAE, serious adverse event; SD, stable disease; TNBC, triple negative breast cancer. Clinical trial numbers (NCT) available from: http://clinicaltrials.gov.

### Farletuzumab

A mAb that has been widely studied in ovarian and lung cancers is farletuzumab, (MORab003). It is a fully humanized IgG1 antibody specific for FRα. Farletuzumab does not prevent binding of folate to its receptors, nor does it inhibit the transport of folate into the cell *via* the receptor. Instead, *in vitro* studies have demonstrated that farletuzumab exhibits activity against FRα-expressing tumor cells by a number of mechanisms. These include tumor cell killing by antibody-dependent cellular cytotoxicity (ADCC) and complement-dependent cytotoxicity (CDC), sustained tumor cell autophagy, and inhibition of Lyn kinase substrate phosphorylation [100–104].

Farletuzumab was first given as monotherapy in a phase I trial in 25 patients with platinum-resistant ovarian cancer (NCT00428766). No dose-limiting toxicities (DLTs) were observed, and dose escalation was continued to a maximum 400 mg/m^2^ dose [105]. Safety of farletuzumab, in combination with carboplatin and liposomal doxorubicin, was then demonstrated in a phase Ib study in patients with platinum-sensitive ovarian cancer (NCT01004380) [106]. In a subsequent phase II study, patients with the same disease were given farletuzumab combined with carboplatin and a taxane, followed by farletuzumab maintenance therapy (NCT00318370). Response rates comparing favorably with historical controls were observed. Overall in these trials, farletuzumab was well tolerated as a single-agent, and did not seem to confer additive toxicity when combined with chemotherapy [107].

Following these promising results, a large phase III trial in patients with platinum-sensitive recurrent ovarian cancer was carried out (NCT00849667). In this trial, farletuzumab, in combination with carboplatin and a taxane, was compared to carboplatin/taxane treatment alone. The primary endpoint of improved PFS was not met, however subsequent analysis suggested an improved PFS in some patient subgroups given higher doses, and in those with lower CA125 levels [28]. Farletuzumab in combination with paclitaxel was also compared to paclitaxel alone in patients with platinum-resistant ovarian cancer in a second phase III study (NCT00738699), but this trial was discontinued early for futility.

Given that patients with high FRα-expressing tumors may derive greater benefit from farletuzumab therapy than those with low expression in tumors, future trials may involve the stratification of patients based on their tumor FRα expression. In fact, the manufacturer of farletuzumab has announced the development of a diagnostic assay to better identify patients with high FRα expression [108]. Furthermore, a phase II study comparing farletuzumab, in combination with carboplatin and paclitaxel, or with carboplatin and liposomal doxorubcin, is currently recruiting patients with platinum-sensitive ovarian cancer and low CA125 (NCT02289950). Another phase II trial will be conducted in patients with FRα-positive TNBC who have low serum levels of CA125 [109].

The clinical efficacy of farletuzumab, in combination with platinum-containing chemotherapy has also been evaluated in non-small-cell lung cancer. This phase II study (NCT01218516) enrolled 130 patients with stage IV adenocarcinoma of the lung and has now completed, although results are yet to be published.

### MOv18 IgG1

The MOv18 IgG1 murine monoclonal antibody was generated by immunization of mice with a surgical specimen of human ovarian carcinoma [32]. The FRα-specific variable regions of the resultant antibody were cloned, and the murine γ1-heavy chains and κ-light chains were subsequently replaced with their human equivalents to engineer a chimeric version of MOv18 IgG [110]. Previous clinical studies of MOv18 IgG (either murine or chimeric) administered to ovarian cancer patients have suggested therapeutic benefit with no overt toxicity.

The first clinical administration of MOv18 IgG1 was as a radiolabeled murine antibody (^131^I-MOv18 IgG1) in 1991. The main aim of this study was to investigate the feasibility of radioimmunoscintigraphy (RIS, the administration of a radiolabelled antibody against a tumor surface marker, for the purpose of imaging the tumor and any metastases). Another objective was to evaluate biodistribution of MOv18 IgG1 for further therapeutic applications [60]. A total of 30 patients with ovarian carcinoma were given ^131^I-MOv18 IgG1 either intravenously (*i.v.*) (*n* = 20) or intraperitoneally (*i.p.*) (*n* = 10). High tumor uptake, a good tumor to background ratio and low non-specific uptake in non-affected organs were observed, with *i.p.* administration superior to *i.v.* administration. These findings suggested that MOv18 IgG1 represented a promising mAb for RIS in ovarian cancer.

Subsequently the same ^131^I-radiolabelled murine antibody was administered as *i.p.* radioimmunotherapy to 16 ovarian cancer patients with minimal residual disease [111]. Efficacy was observed, with 5 complete responses and 5 patients demonstrating stable disease. In addition the toxicity was reported to be low with only one patient showing mild and transient bone marrow suppression. However, human anti-mouse antibody (HAMA) production was demonstrated in 94% of patients.

In order to reduce HAMA, a chimeric MOv18 IgG1 was generated, and radiolabeled with either ^131^I or ^125^I, before administration to 24 patients with ovarian carcinoma [33]. Six days after injection, the antibody was demonstrated to localize well in ovarian carcinoma tissue with a mean tumor to normal tissue ratio of 6.7, indicating a prolonged accumulation in the tumor relative to normal tissues. In view of these positive findings, the safety of a single *i.v.* infusion of increasing doses (5-75mg) of chimeric MOv18 IgG1 was subsequently evaluated in a phase I study of 15 ovarian carcinoma patients [112]. Administration of MOv18 IgG1 was demonstrated to be safe with no significant changes in hematological, biochemical, or urine profiles detected. However, at doses of 50 mg and above all patients experienced minor (World Health Organization, WHO grade 2) side effects, including fever, headache, and nausea/vomiting. Interestingly, no human anti-chimeric antibody (HACA) response was detected up to 12 weeks post injection. These findings suggested that targeting of FRα with chimeric MOv18 IgG1 was safe and that this mAb represented a promising therapeutic for the treatment of ovarian carcinoma.

Since ovarian cancer is mainly limited to the peritoneal cavity, locoregional delivery of therapeutics can be an option. Therefore the influence of the route of administration (*i.v.* or *i.p.*) of radiolabeled chimeric MOv18 IgG1 was investigated in two studies [61, 113]. In the first study, ^131^I-MOv18 IgG1 was administered to 12 patients with ovarian cancer. Scintigraphic images after *i.p.* administration showed better accumulation in ovarian cancer lesions compared with after *i.v.* administration. Furthermore, there was no normal organ toxicity. This study concluded that the *i.p.* route of administration was safe and seemed to be preferable to *i.v.* administration [113]. In the second study 15 patients received chimeric MOv18IgG1 labeled with ^131^I, ^125^I and ^123^I, either *via* the *i.p.* or *i.v.* route. No adverse events and no HACA response were reported. Furthermore, in contrast to the previous study, no advantage could be demonstrated for the *i.p.* route of administration with respect to tumor uptake; however, it was suggested that it may be the preferred route with respect to bone marrow toxicity since the area under the curve (AUC) was significantly lower for the *i.p. versus* the *i.v.* route [61].

In summary, numerous clinical studies conducted with murine or chimeric MOv18 IgG1 to date suggest that targeting the tumor antigen FRα with a therapeutic mAb is safe, with minimal toxicities observed across all studies. However, some studies have suggested superior tumor targeting when the antibody was administered locoregionally to the tumor, perhaps indicating relatively poor tumor penetrance of MOv18 IgG1 when administered *via* the *i.v.* route. This suggests the possible benefit of undertaking antibody engineering strategies, including re-engineering approaches or changing the isotype of the antibody, with the aim of improving bioactivity, potency or tumor penetrance aimed at enhancing antibody efficacy.

### MOv19 and derivatives

The mAb MOv19 was selected from the same fusion from which MOv18 was derived. MOv19 and LK26 (the murine mAb from which the fully humanized Farletuzumab was derived) recognize the same or overlapping epitopes on FRα, but independent from the epitope recognized by MOv18, as detected by competition assay in Biacore analysis and ELISA [32, 114].

Chimeric versions of MOv18 and MOv19 IgG antibodies, similarly to Farletuzumab, mediate both ADCC [115] and low levels of CDC [116] *in vitro*. However, when the two chimeric mAbs were mixed, a significant increase in tumor cell killing was observed of up to 50%. This value increased to 70% after neutralization of CD46 and CD59 (membrane C regulatory molecules) without an appreciable change of ADCC. These results suggest that complement can contribute to the killing of ovarian carcinoma cells induced by the mixture of cMOv18 and cMOv19.

Several different derivatives of MOv19 have been evaluated in different therapeutic applications. Completely human Fab fragments against FRα were produced by using phage display and among them one, named C4, exhibited good specificity [117]. The human C4 in scFv format, after adequate optimization, resulted in CAR redirection [118].

By applying epitope imprinting selection [119], a method that enables isolation of antibodies with the same specificity of a pre-existing antibody, a human Fab (AFRA5), recognizing a FRα epitope overlapping with that of MOv19, was identified [120]. After optimization of the lead reagent, a chemical dimer, named AFRA-DFM5.3, was considered suitable for further *in vivo* preclinical evaluation in the perspective of a clinical use. In fact, an antibody fragment, in a dimer format that stabilizes binding as soon as the antigen-antibody complex is formed on the target tumor, might be the reagent of choice for *i.p.* radioimmunotherapy of ovarian cancer because of its relatively small size, which should favor tumor penetration and fast clearance. Due to its fast and high tumor uptake, ^131^I-AFRA-DFM5.3 resulted in more than 50% of treated animals cured in a preclinical intraperitoneal model [121]. The human origin of AFRA-DFM5.3 and its efficacy when delivered locoregionally as an ^131^I reagent, together with evidence of the feasibility and acceptable toxicity profile of ovarian cancer treatment with anti-FR mAbs, could provide the basis for rational design of new therapeutic modalities.

### MOv18 IgE

MOv18 IgE is an anti-FRα chimeric IgE antibody, engineered from the variable heavy and light chain regions of MOv18 IgG1 in order to investigate the hypothesis that IgE antibodies may offer advantages as immunotherapeutic agents against cancer compared to their IgG counterparts [122]. The rationale for using IgE antibodies against cancer stems from the unique properties of this class, which make it a critical contributor to allergic and parasitic immune responses. These properties include the exceptionally high affinity of IgE to its Fc receptors (FcεRI and CD23), the lack of an inhibitory IgE receptor, and the significantly longer half-life of IgE in tissues compared to IgG1, which results in improved local retention of IgE. IgE antibodies are thus able to confer potent immune responses by activating tumor-resident immune effector cells [123].

To date, a number of *in vivo* models have been used to evaluate the efficacy of MOv18 IgE. Firstly, immunodeficient (SCID) mice challenged subcutaneously (*s.c.*) with FRα-expressing human ovarian carcinoma cells, were treated with human peripheral blood lymphocytes (PBLs) and MOv18 IgE or IgG1 antibodies *i.v.* [122]. Secondly, a xenograft mouse model was set-up using patient-derived FRα-expressing human ovarian carcinoma, and mice were given human peripheral blood mononuclear cells (PBMCs) *i.p.* with MOv18 IgE or IgG1 antibodies [124–126]. MOv18 IgE afforded superior anti-tumor efficacy and animal survival, compared with its IgG1 counterpart, in both models.

These studies also provided evidence that MOv18 IgE efficacy may be mediated *via* anti-tumor effector cell functions *in vivo*: addition of PBMCs was required for the observed protective effect and this efficacy was ablated upon depletion of monocytes; monocytic infiltration was observed in tumor sections from mice treated with MOv18 IgE; and large areas of tumor necrosis were detected following MOv18 IgE treatment [122, 126]. MOv18 IgE-mediated tumor cell killing by human monocytes and eosinophils was also confirmed *in vitro* by ADCC/ADCP [126, 127]. Emerging evidence point to the capacity of IgE antibodies to activate host immune effector cells against cancer and support its anti-tumor efficacy [128–131]. This agent is now being translated for clinical testing in FRα-expressing carcinomas.

### T cell activating strategies

The possibility to combine the antibody specificity with the potency of T cell weapons to treat tumors, independently from the T cell receptor (TcR)-defined specificity, and expression of human leukocyte antigen (HLA) on cancer cells, has been explored and specific reagents for T cell retargeting were developed. Among different approaches, the most promising are the use of bi-specific antibodies (BsAbs), T-cell engineering to create chimeric antigen receptors (CARs), as well as combinations with immune checkpoint inhibitors.

Adoptive transfers of *ex vivo* T cells armed with the BsAb OC/TR (MOv18 x anti-CD3) [132] or of CAR-engineered T cells recognizing FRα [133] (Table 2) were among the first clinical attempts with these therapeutic tools.

In a phase I/II study, among the 28 ovarian cancer patients treated intraperitoneally with OC/TR-armed activated T cells and IL-2, response to treatment could be assessed in 26 patients by explorative laparotomy. The overall intraperitoneal response rate was 27%. The complete responses seen in three patients lasted 26 months in one patient, 23 months in the second, and 18 months in the third [132]. On the contrary the outcome of the phase I clinical trial with a FRα-specific CAR T cell treatment was discouraging with no response observed in any patient and the presence of significant toxicities [133]. It is noteworthy that both trials have been conducted using the anti-FRα murine monoclonal antibody MOv18 (see further discussion of this antibody below) and that in the OC/TR trial almost all the patients developed a HAMA response that precluded further treatment [134] and in the CAR trial an inhibitory factor developed in the serum of three of six patients tested over the period of treatment, which significantly reduced the ability of gene-modified T cells to respond against FRα positive tumor cells [133].

Even if no clinical results are at present available with these anti-FRα tools, advances in protein engineering and increased knowledge in T cell biology have enabled the rise of both BsAbs and CARs from inefficient first generation reagents to promising molecules for cancer treatment. In particular, first generation CARs failed because of poor T cell expansion and adverse effects; new second and third generation CARs have solved at least in part these problems and good pre-clinical *in vivo* results, *i.e.* tumor regression, longer persistence in circulation and better localization towards the tumor, have been obtained with MOv19-based CAR constructs containing the co-stimulatory motif of CD137 or CD27 [118, 135].

Another approach, which has recently seen increased interest, is the use of antibodies targeting immune cell checkpoints that negatively regulate anti-tumor immunity [136]. Ipilimumab targets cytotoxic T lymphocyte-associated antigen 4 (CTLA-4), which is involved in an alternative interaction between T cells and antigen-presenting cells (APCs) that inhibits T cell effector functions. Similarly, nivolumab and pembrolizumab target the interaction between programmed cell death receptor 1 (PD-1), which is expressed on activated effector cells such as T cell, B cells and other myeloid cells, and its ligand PD-L1, which is expressed on tumor cells and APCs. Treatment with these immune checkpoint-targeting antibodies prevents the attenuation of T cell activation and thereby enhances anti-tumor immunity. Ipilimumab and nivolumab have shown significant efficacy in patients with metastatic melanoma and are now widely approved for treatment of this solid tumor. Clinical trials in patients with other tumor types, including ovarian, lung and breast, are now underway and demonstrating promising results [137–140]. Thus, the combination of checkpoint inhibitors and FRα-targeting strategies may well lead to greater therapeutic success.

## CONCLUSIONS

Insights into tumor expression and distribution of FRα and its emerging roles in cancer growth and metastasis are now focusing renewed interest on this tumor-associated antigen as a potential target and tumor marker for solid tumours such as ovarian, lung and basal breast cancers. There is an unmet need in these malignancies, associated with particularly poor prognosis, for further treatment options as well as biomarkers (predictive and prognostic). Future focus on FRα offers real potential for the development of targeted cancer therapies. Past and ongoing clinical evaluations of FRα-targeted therapies, such as vintafolide and farletuzumab, allow for cautious optimism, but suggest that both improved patient selection and optimised modes of action are still needed.

Low levels of FRα expression in most normal tissues predict a low probability of significant toxicity for these agents, supported by clinical experience so far with treatment strategies targeted using folate and antibodies. FRα expression in tumor may serve as both a predictive marker to guide patient selection for such strategies, and also as a potential prognostic indicator. Furthermore, serum detection of circulating soluble antigen levels might also serve as an effective diagnostic or prognostic marker, and potentially an indicator of treatment response. These possible uses still require further study in FRα-expressing malignant indications.

A range of novel immunotherapeutic modalities, including vaccines, oncolytic viruses, monoclonal antibodies and adoptive T-cell strategies, with novel mechanisms of action, are in preclinical or clinical studies. Perhaps those agents with enhanced immune activating properties, more resistant to tumor-associated immune suppressive mechanisms, may in future become effective strategies against FRα-expressing tumors.
